# A novel small-molecule inhibitor of influenza A virus acts by suppressing PA endonuclease activity of the viral polymerase

**DOI:** 10.1038/srep22880

**Published:** 2016-03-09

**Authors:** Shuofeng Yuan, Hin Chu, Kailash Singh, Hanjun Zhao, Ke Zhang, Richard Y. T. Kao, Billy K. C. Chow, Jie Zhou, Bo-Jian Zheng

**Affiliations:** 1 Department of Microbiology, The University of Hong Kong, Hong Kong SAR, China; 2 School of Biological Sciences, The University of Hong Kong, Hong Kong SAR, China

## Abstract

The RNA-dependent RNA polymerase of influenza A virus comprises conserved and independently-folded subdomains with defined functionalities. The N-terminal domain of the PA subunit (PA_N_) harbors the endonuclease function so that it can serve as a desired target for drug discovery. To identify a class of anti-influenza inhibitors that impedes PA_N_ endonuclease activity, a screening approach that integrated the fluorescence resonance energy transfer based endonuclease inhibitory assay with the DNA gel-based endonuclease inhibitory assay was conducted, followed by the evaluation of antiviral efficacies and potential cytotoxicity of the primary hits *in vitro* and *in vivo*. A small-molecule compound ANA-0 was identified as a potent inhibitor against the replication of multiple subtypes of influenza A virus, including H1N1, H3N2, H5N1, H7N7, H7N9 and H9N2, in cell cultures. Combinational treatment of zanamivir and ANA-0 exerted synergistic anti-influenza effect *in vitro*. Intranasal administration of ANA-0 protected mice from lethal challenge and reduced lung viral loads in H1N1 virus infected BALB/c mice. In summary, ANA-0 shows potential to be developed to novel anti-influenza agents.

The continuous zoonotic circulation and re-assortment potential of influenza A viruses in nature have been posing an enormous public health threat to humans^1^,^2^,^3^. Due to the limitations of vaccines, which are about 0.5–1 year lagged because of the rapid mutations of the virus genes, antiviral drugs represent an important approach to combat human influenza diseases, particularly in the early stage of influenza outbreaks and pandemics^4^. Two classes of antiviral drugs, targeting the matrix protein 2 (M2) and the neuraminidase (NA) protein of influenza virus, have been approved for clinical treatment. However, the prevalence of drug-resistant strains, e.g. in seasonal H3N2, pandemic H1N1, avian influenza H5N1 and H7N9, has undermined their clinical benefits to certain extent^5^,^6^,^7^. In this regard, new antivirals with cross-protection are highly desired. Biological and structural investigations of the functional domains of these subunits have broadened the target reservoir for drug screening. With the wealth of knowledge from these studies, identification of small-molecule inhibitors that specifically disrupt the polymerase assembly or abrogate polymerase activity has emerged as an innovative and promising approach^8^. Importantly, the domains that are responsible for the above-mentioned functions are fairly conserved^9^. Therefore, drugs with cross-subtype antiviral effects are likely to be discovered.

The RNA-dependent RNA polymerase (RdRp) of influenza A virus, which consists of the PB1, PB2 and PA subunits, serves as the hub for viral transcription and replication^10^. The viral gene transcription is primed by short-capped oligonucleotides that are cleaved from host cell pre-mRNAs by PA endonuclease activity, a process known as ‘cap-snatching’^11^. The N-terminal domain of PA subunit (PA_N_) has been confirmed to accommodate the endonuclease activity residues, which is highly conserved among subtypes of influenza A virus and is able to fold functionally *in vitro*^12^,^13^. Importantly, substitutions in this functional domain, particularly the catalytic residues, were expected to considerably reduce the viral fitness^14^,^15^. In this regard, the emergence of resistant virus mutants induced by PA_N_ antivirals may be significantly delayed.

Amino acid residues in the PA_N_ region play critical roles in endonuclease activity, protein stability and vRNA promoter binding^13^. One recent study revealed that PA_N_-derived peptides effectively inhibited ribonucleoprotein (RNP) activity via suppression of RNP expression, suggesting that this region itself might be potential influenza virus inhibitors^16^. Determination of the PA_N_ crystal structure^12^,^13^ has paved the path for the development of endonuclease inhibitors, including 2,4-dioxobutanoic acid derivatives^17^,^18^,^19^,^20^, flutimide derivatives^17^,^18^,^21^, 3-hydroxyquinolin-2(1*H*)-ones and 3-hydroxypyridin-2(1*H*)-ones^22^,^23^, as well as tetramic acid derivatives^24^. These endonuclease inhibitors were screened by computational modeling^25^,^26^,^27^ or identified through the measurement of nucleic acid hydrolysis^28^,^29^ or cap-snatching activity^30^. Recently, a fluorescence polarization assay has been established for screening small-molecule binders of PA_N_, through which a group of endonuclease inhibitors were identified^31^. However, PA_N_-targeted inhibitor that possesses promising clinical potential is rare. In an attempt to facilitate the discovery of PA endonuclease inhibitors, we performed a screening that integrated fluorescence resonance energy transfer (FRET) based endonuclease inhibitory assay^29^ with DNA gel-based endonuclease inhibitory test^12^. FRET relies on the distance-dependent energy transfer between two labeled molecules^32^, it has been successfully applied in high-throughput screening (HTS) to identify inhibitors of numerous targets^33^,^34^. In this report, we described the optimization, validation and application of the FRET-based endonuclease assay for screening of a chemical library^35^ with 950 small-molecule compounds. A number of influenza A inhibitors were identified. We then proceeded to conduct various experiments to investigate the antiviral mechanism. Notably, an inhibitor(5Z)-2-[2-(2-oxoindol-3-yl)hydrazinyl]-5-(2-oxo-1H-indol-3-ylidene)-1,3-thiazol-4-one, designated ANA-0, exhibited potent and cross-subtype antiviral effects with high selectivity index.

## Results

### Establishment of FRET-based endonuclease inhibitory assay

**Figure 1:**
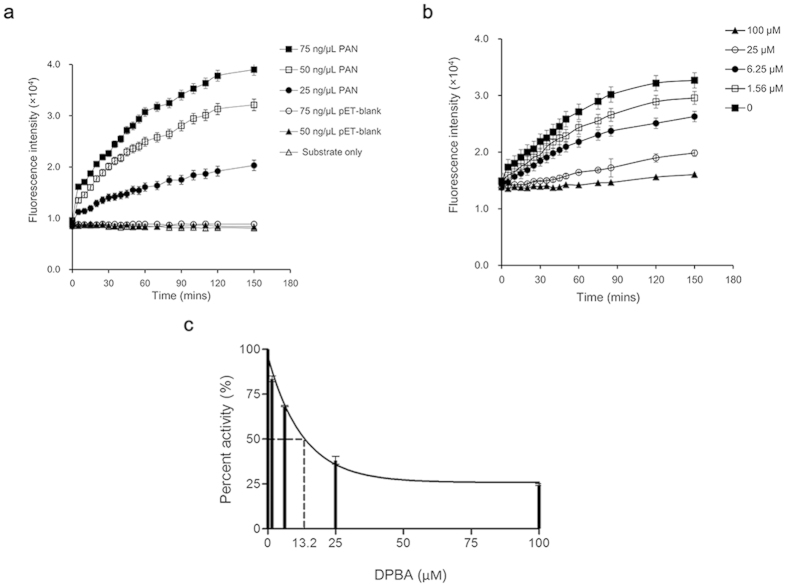
Detection of PA_N_ endonuclease activity by FRET-based assay. The fluorescence intensity of each reaction was recorded at indicated time-points. (**a**) PA_N_ of depicted concentrations were reacted with 200 nM of the dual-labeled probe, while pET-blank protein and substrate only were included as a mock-purified enzyme control and a background control, respectively. (**b**) Indicated concentrations of DPBA were incubated with a mixture of 75 ng/μl PA_N_ and 200 nM probe. Fluorescence intensities were recorded and results are represented. Reactions of each condition were done in triplicate. (**c**) Fitting of DPBA dose-response curve to yield IC_50_ by nonlinear regression model. Results are shown as the mean values ± SD.

The FRET-based endonuclease inhibitory assay was developed for screening of PA endonuclease inhibitors. To detect the endonuclease activity of PA_N_, we first demonstrated a dose-dependent increase of fluorescence intensity over time upon PA_N_ addition to the dual-labeled probe (Fig. 1a). The fluorescence signals reached a plateau at 2 h post-reaction. Compared with the baseline, a maximal of 4-fold signal increase could be detected with the input of 75 ng/μl of PA_N_. In contrast, the mock purified enzyme protein (i.e. pET-blank protein), regardless of the amounts of addition (75 and 50 ng/μl), exhibited similar readouts as that of the background control (i.e. substrate only). The result suggested that the purified PA_N_ indeed maintained the endonuclease activity. To validate the specificity, fluorescence signals were recorded in the presence or absence of a known PA endonuclease inhibitor DPBA^36^. At the fixed concentration of 75 ng/μl of PA_N_, a dose-dependent inhibition was detected, in which higher concentrations of DPBA resulted in lower fluorescence intensities (Fig. 1b). The result suggested that the PA_N_ endonuclease activity was specifically inhibited by DPBA. The inhibitory constant (Ki) and 50% inhibitory concentration (IC_50_) of DPBA in this assay were estimated as 12.8 μM and 13.2 μM (Fig. 1c), respectively. These results were in the range of those reported previously by others^12^,^20^,^29^ and supporting that a sensitive and specific FRET-based endonuclease inhibitory assay was established for the effective screening of endonuclease inhibitors.

### Identification of antiviral compounds

**Figure 2:**
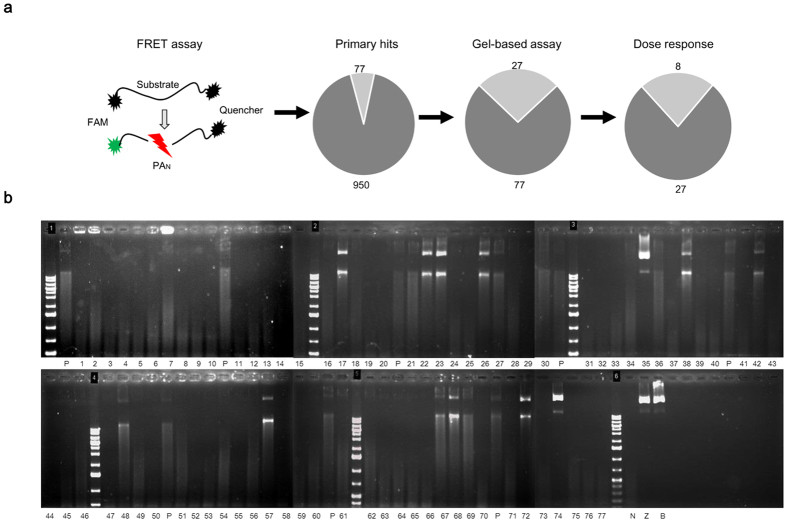
Identification of compounds by their inhibitory activity of endonuclease. (**a**) Schematic diagram of FRET-based assay and attrition rates of compounds from primary screening, gel-based endonuclease inhibitory assay and dose-response analysis. (**b**) Screening of compounds with gel-based endonuclease inhibitory assay. The single-strand circular DNA M13mp18 was used as the substrate. The substrate control (lane Z), buffer control (lane B) and no-compound control (lane N) were included as negative controls. DPBA (10 μM) was taken as a positive control (lane P) and was carried out every 10 candidate compounds for reference comparison. In each reaction, 10 μM of individual compound was mixed with 1 μM PA_N_ and subsequently incubated with 0.2 μg M13mp18 substrate in 10 μl volume. The images were based on DNA agarose gels and ethidium bromide staining.

**Figure 3:**
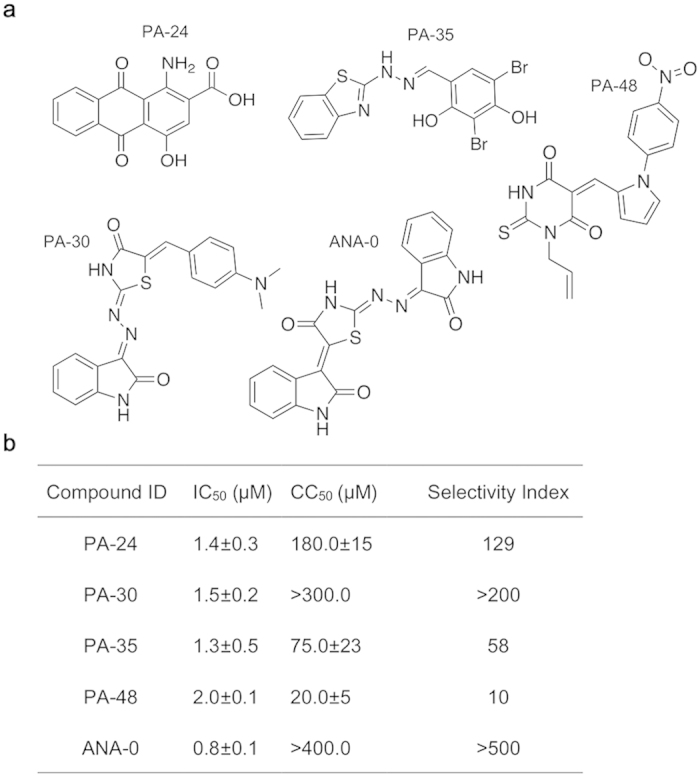
Chemical structures and selectivity indexes of antiviral compounds. (**a**) Chemical structures of antiviral compounds PA-24, PA-30, PA-35, PA-48 and the PA-30′s analog ANA-0 are shown. (**b**) Selectivity index of each compound was calculated by CC_50_/IC_50_. For CC_50_ determination, the highest concentrations of the compounds PA-30 and ANA-0 cannot be determined in MTT assay due to solubility limitations.

As shown in Fig. 2a, compounds in the library were first screened at a fixed concentration of 10 μg/ml (25~50 μM) using the established FRET-based endonuclease inhibitory assay. A total of 77 compounds displayed the decreased fluorescence intensities that >50%. We then performed the DNA-gel based endonuclease inhibitory analyses to exclude false-positive results that might be produced by fluorescence interference from the compound itself (Fig. 2b). It was demonstrated that the PA_N_ was endonuclease active as the M13mp18 substrate was largely diminished under the PA_N_ digestion (lane N), in contrast, the substrate remained intact in both the substrate and the buffer controls (lane Z and B). As a result, 27 compounds were defined as ‘active’ by showing stronger endonuclease inhibitory effect than that of 10 μM DPBA (lane P). Next, a dose-response analysis was performed to identify the compounds that could consistently suppress the PA_N_ endonuclease activity. In this experiment, a total of 8 compounds were selected due to their endonuclease inhibitory activities in a concentration-dependent manner. Subsequently, a cell-based secondary screening was applied to search inhibitors with antiviral activities. Four compounds, namely PA-24, PA-30, PA-35 and PA-48 (Fig. 3a), were identified to reduce the plaque number in a dose-dependent manner and were regarded as antiviral-effective compounds. The selectivity index of individual compound, defined by the ratio of 50% cellular cytotoxicity concentration (CC_50)_ over IC_50_, was determined to prioritize these 4 compounds. The results showed that PA-30 possessed the highest selectivity index (>200, Fig. 3b). Based on the structural properties of compounds PA-24, PA-30, PA-35 and PA-48, structural similar analogs with apparently good water solubility (logSw >−4.75) and low molecular weight (MW <425)^37^ were purchased from commercial sources. A total of 14 analogs were obtained, whose selectivity index was scored individually. Compound ANA-0 (Fig. 3a), an analog of PA-30, exhibited the best selectivity index that >500 and was chosen for further evaluation.

We then conducted a multi-cycle virus growth assay to evaluate the antiviral efficacies of PA-30 and ANA-0. Both compounds displayed dramatic anti-H1N1 effects with 2–3 log reduction in supernatant viral titer (supplementary Fig. S2), while ANA-0 showed higher selectivity index than that of PA-30 (Fig. 3b). Thus, we further evaluated the cross-subtype antiviral effect of PA-30 and ANA-0 *in vitro*.

### ANA-0 provided cross-subtype protection against influenza A virus infections *in vitro*

**Figure 4:**
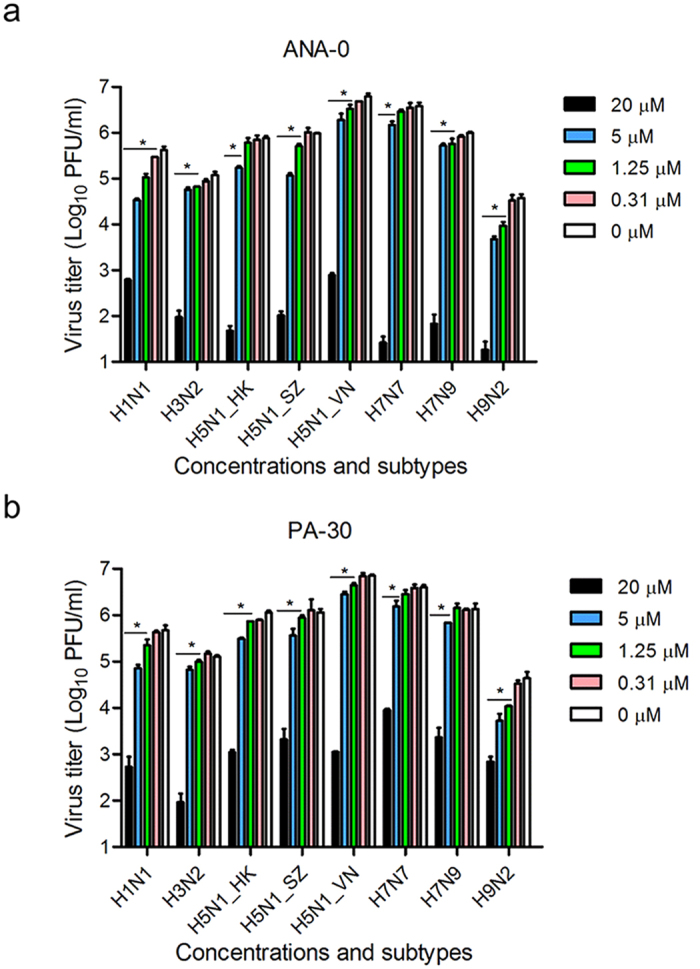
*In vitro* antiviral activity of ANA-0 and PA-30. Antiviral activities of ANA-0 (**a**) and PA-30 (**b**) were determined by plaque assays. MDCK cells were infected with different strains of virus as shown, at MOI of 0.002. One hour after virus inoculation, the inoculum was removed and replaced by fresh MEM medium containing serial-diluted compound. The cell-free supernatants were collected at 24 h post-infection and titrated by standard plaque assay. The experiments were carried out in triplicate and repeated twice. Data are represented as mean values + SD. Differences between various concentrations treatments were compared and analyzed using a one-way ANOVA. *indicates *p* < 0.05 as compared to mock-treated group.

Since the sequence of PA_N_ is highly conserved among viral strains (supplementary Fig. S1), we speculated that ANA-0 and PA-30, which were considerably effective against H1N1 virus infection (supplementary Fig. S2), might be capable to provide cross-protection against the infections of other subtypes of influenza virus. To this end, cross-subtype antiviral effects of both agents were tested against infections of H3N2, H5N1, H7N7, H7N9 and H9N2 viruses in cell cultures. The results showed that both ANA-0 and PA-30 inhibited viral replication of all tested subtypes of influenza virus in a dose-dependent manner (Fig. 4). At 20 μM, ANA-0 suppressed the virus replication of all tested subtypes by more than 3 logs, whereas different subtypes of the virus exhibited variable sensitivities to ANA-0 (Fig. 4a). For example, ANA-0 showed superior antiviral effect against H1N1 and H9N2 virus infection with IC_50_s lower than 1 μM. In contrast, it required 5-fold higher concentrations to achieve the similar level of inhibition against H3N2 and H7N9 viruses’ infections, while IC_50_s of ANA-0 against infections of H5N1 and H7N7 viruses were around 2.5 μM. PA-30 exhibited similar pattern of antiviral activity with that of ANA-0 (Fig. 4b).

### ANA-0 inhibited virus growth *in vivo*

**Figure 5:**
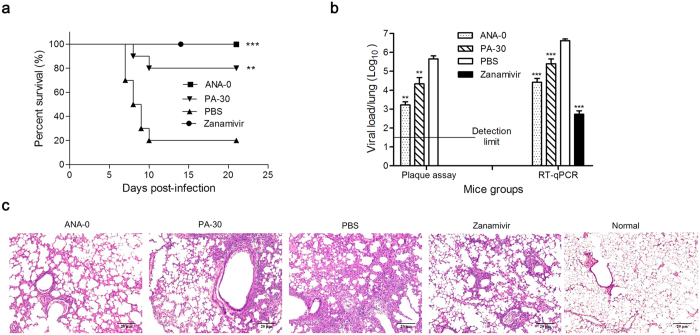
*In vivo* antiviral activity of ANA-0 and PA-30. (**a**) Mice (10 per group) infected with LD_80_ (500 PFU/mouse) of mouse-adapted A/HK/415742Md/09 H1N1 virus were treated with 2 mg/kg/day of ANA-0 or PA-30 or zanamivir or PBS by intranasal administration. Treatments started at 6 h after virus challenge and continued for 6 doses in 3 days (2 doses/day). Difference between groups were compared and analyzed using Log-rank (Mantel-Cox) test. ***indicates *p* < 0.001 and **indicates *p* < 0.01 as compared to PBS-treated group. (**b**) Four mice from each group were euthanized at day 4 post-infection and lungs were collected for detection of viral loads by plaque assay (detection limit: 1:50) and RT-qPCR. The plaque was undetectable in the lung samples of zanamivir-treated mice. The results are presented as the mean values + SD. Differences between groups were compared and analyzed using a one-way ANOVA. ***indicates *p* < 0.001 and **indicates *p* < 0.01 as compared to PBS-treated group. (**c**) Histopathologic changes in mouse lung tissues collected at day 4 post-infection. Representative histologic sections of the lung tissues from the mice were stained with H&E (magnification: × 100). Less inflammatory infiltrate and thickening of the alveolar septum (as alveolar damage) are shown in samples from mice treated with ANA-0, PA-30 and zanamivir as compared to that from PBS-treated mice.

To assess the *in vivo* antiviral effect of ANA-0, mice challenged with LD_80_ of mouse-adapted H1N1 virus were treated with ANA-0 or PA-30 or zanamivir or PBS. As shown in Fig. 5a, all mice that received intranasal treatment with 2 mg/kg/day ANA-0 or 2 mg/kg/day zanamivir survived (*p* = 0.0003), while 2 mg/kg/day PA-30-treated group showed 80% survival rate (*p* = 0.0049); in contrast, 80% mice died in PBS-treated group. Four mice were euthanized from each group on the 4th day after infection and their lungs were tested for virus titer by plaque assay and RT-qPCR. The results showed that ANA-0-treated group exhibited significant reduction of viral loads in the lung tissues as compared with the control group (*p* = 0.0013 by plaque assay and *p* = 0.0006 by RT-qPCR), while PA-30-treated group inhibited virus growth by more than 1 log (*p* = 0.0032 by plaque assay and *p* = 0.0008 by RT-qPCR). Histopathologic examination further showed that the alveolar damage and interstitial inflammatory infiltration in lung tissues of the mice treated by ANA-0 or PA-30 were much ameliorated than that of those treated by PBS (Fig. 5c). The results demonstrated that ANA-0 could effectively inhibit the influenza virus propagation *in vivo*.

### ANA-0 inhibited the viral transcription

**Figure 6:**
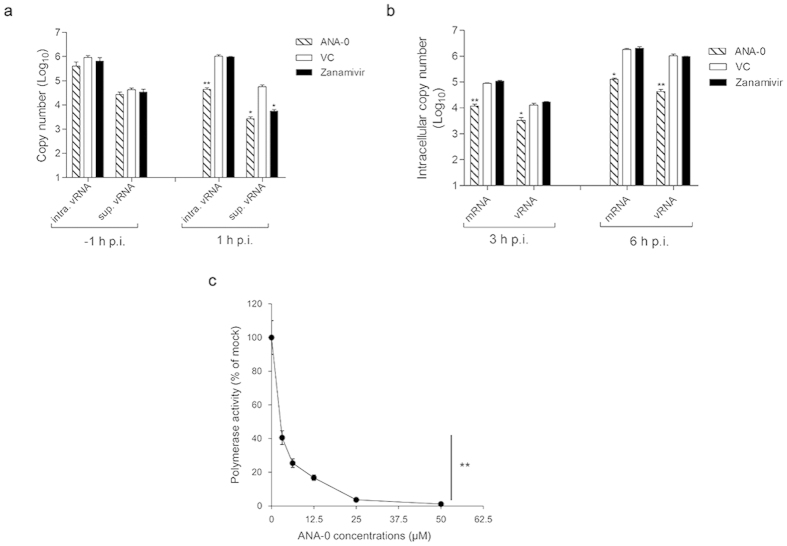
Antiviral mechanism of ANA-0. (**a**) Intracellular viral RNA (intra. vRNA) and supernatant viral RNA (sup. vRNA) were quantified in a time-of-addition assay. MDCK cells were inoculated with influenza H1N1 virus (MOI = 2), while ANA-0 (20 μM) or zanamivir (100 μM) was added at the time of virus absorption (−1 h) and then removed or at 1 h post-infection (p.i.) and then maintained in the medium. vRNA copies in the cells or supernatants were determined at 6 h p.i. (**b**) MDCK cells were infected with influenza H1N1 virus with MOI of 2 for 1 h. The cells were washed and maintained in the medium containing ANA-0 (20 μM), or zanamivir (100 μM) or mock-treated (VC) thereafter. Intracellular virus-specific mRNA and vRNA were quantified at 3 or 6 h p.i. (**c**) Inhibitory effect of ANA-0 to viral polymerase activity was tested by a mini-replicon assay. 293 T cells were transfected with plasmids encoding PB1, PB2, PA, NP genes, a firefly luciferase reporter-gene plasmid and an eGFP plasmid for transfection efficiency normalization. Indicated concentrations of ANA-0 were added at 5 h post-transfection. Luminescence and fluorescence were determined at 24 h post-transfection, respectively. The experiments were carried out in triplicate and repeated twice. The results are presented as mean values ± SD. Differences between groups were compared and analyzed using a one-way ANOVA. **indicates *P* < 0.01 as compared with the mock-treated control.

To verify the antiviral mechanism of ANA-0, we first determined which phase of virus life cycle was interrupted by ANA-0. As shown in Fig. 6a, ANA-0 did not exert antiviral efficacy when it was added during virus absorption (i.e. -1 h p.i.) and subsequently removed after virus entry. A significant decrease of viral RNAs (vRNAs), both intracellular (*p* = 0.0074) and in the supernatant (*p* = 0.0183), were detected when ANA-0 were maintained in the culture medium after virus entry (i.e. 1 h p.i.). In contrast, addition of zanamivir reduced the vRNA in the supernatant but not inside the cells (Fig. 6a). The results supported that ANA-0 interfered the virus life cycle at stages after virus internalization but prior to budding. We further investigated which step of viral replication was interfered. As shown in Fig. 6b, ANA-0 treatment reduced the viral mRNA production at either 3 or 6 h post-infection, suggesting that ANA-0 inhibited the viral transcription. Since the primary viral transcription occurs before viral genome replication^38^, the treatment of ANA-0 also resulted in subsequent decrease of vRNAs in cell lysates (*p* = 0.0412 for 3 h p.i. and *p* = 0.0067 for 6 h p.i. (Fig. 6b)). The results indicated that ANA-0 disrupted the transcriptional stage of virus life cycle so that inhibited viral replication. We then conducted a mini-replicon assay to investigate the inhibitory efficacy of ANA-0 on the influenza polymerase activity. As shown in Fig. 6c, a dose-dependent suppression of luciferase activity was observed, suggesting that the viral polymerase function was impaired in the presence of ANA-0.

### Synergistic antiviral effect of ANA-0 and zanamivir *in vitro*

Since antiviral mechanism of ANA-0 was distinct from the commonly prescribed influenza NA inhibitor zanamivir, we further investigated the potential synergistic antiviral effects between two agents *in vitro*. Fractional inhibitory concentration index (FICI) is one of the popular methodologies for evaluating the nature of drug-drug combination^39^,^40^. The FICI is based on the Loewe additive zero-interaction theory^41^, assuming that a self-drug combination will always be additive, with an FICI of 1; while an FICI lower or higher than 1 indicates synergy or antagonism, respectively, because less or more drug would be required in order to produce the same effect as the drugs alone. In this study, five sets of combinations were conducted and FICI of each was determined. As shown in Table 1, all tested combinations resulted in FICI that <0.5, which suggested the strong synergism existed between ANA-0 and zanamivir. Among the five, binary usage of 0.8 μM ANA-0 and 0.05 μM zanamivir, i.e. combination ratio (IC_50_) 1:1, exerted the best synergistic efficacy (FICI = 0.24) against virus infection (Table 1).

### ANA-0 was predicted to interact with the PA endonuclease pocket

**Figure 7:**
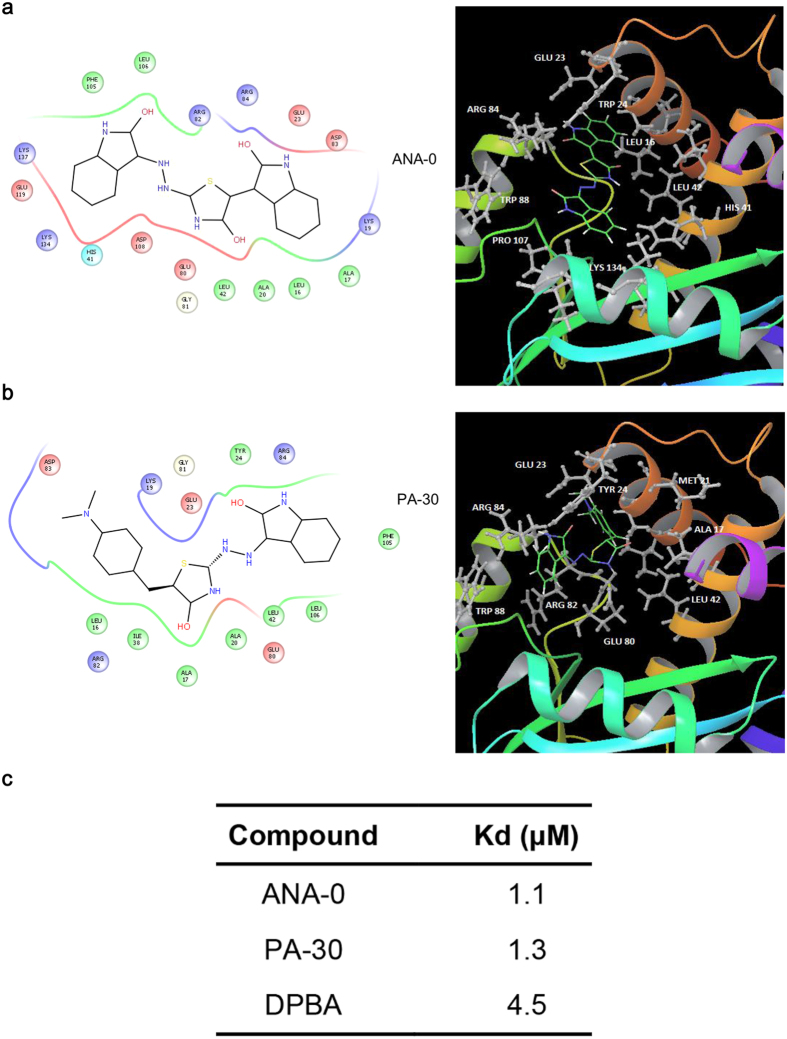
Docking simulation of ANA-0 and PA-30 with influenza PA_N_ domain. Two dimensional analysis (left) and ribbon diagram (right) of the interactions between ANA-0 **(a**) or its parental compound PA-30 (**b**) with PA_N_ are shown. (**a**) ANA-0 is predicted to interact with the endonuclease pocket by contacting the catalytic residues Lys-134, the metal binding residues His-41, Glu-80, Asp-108, Glu-119 and two strictly conserved residues Arg-84 and Lys-137. (**b**) PA-30 is predicted to interact with the residues Ala-20, Leu-42, Glu-80, Gly-81 and Leu-106. In 3D structural analyses, chemical structures of ANA-0 and PA-30 are shown as colored stick models, while interaction amino acid residues are labeled as grey. (**c**) Binding affinity (Kd) of ANA-0 and PA-30 to PA_N_ protein were determined by isothermal titration calorimetry and compared with the reported value of DPBA.

Molecular docking was performed to predict the essential amino acid residues in PA_N_ that were responsible for the interaction with ANA-0 or its parent compound PA-30 (Fig. 7). A parallel study using DPBA as a natural ligand was included. The prediction revealed that ANA-0 bound to the catalytic residues Lys-134, the metal binding residues His-41, Glu-80, Asp-108, Glu-119 and two strictly conserved residues Arg-84 and Lys-137 of PA_N_ structure (Fig. 7a); while PA-30 interacted with the residues of Ala-20, Leu-42, Glu-80, Gly-81 and Leu-106 (Fig. 7b). The predictions suggested that ANA-0 and PA-30 were likely to bind to the PA_N_ endonuclease cavity. In addition, the Kd values of ANA-0 and PA-30 to PA_N_ were experimentally determined to be 1.1 μM and 1.3 μM, respectively (Fig. 7c). In comparison with the reported Kd of DPBA^17^, i.e. 4.5 μM, the result suggested that the identified compounds bound tighter to the PA_N_. Since the predicted interaction amino acids of ANA-0 were key for the PA_N_ endonuclease activity, the results supported that ANA-0 was an endonuclease inhibitor by binding to the enzyme activity sites.

## Discussion

In this study, we performed a systematic screening in a chemical library and identified a novel small-molecule compound with broad-spectrum antiviral activities against influenza A viruses. To establish the screening approach, we initially expressed the N-terminal domain of PA subunit that retained the endonuclease activity (Fig. 1a). Based on the rationale that cleavage of 5′-fluorophore and 3′-quencher labeled probe by endonuclease could be detected by an increase of fluorescence, we then developed a FRET-based endonuclease inhibitory assay for screening small-molecule endonuclease inhibitors (Fig. 1b). In the assay, a DNA probe was designed to avoid the potential interferences of RNase contamination. Also, mock-purified pET-blank enzyme was included as a negative control to exclude potential *E.coli*-DNase influences. Even so, false positive results may be obtained in a FRET-based screening, typically due to compounds which are fluorescent quenchers or due to the inner filter effect^42^, i.e., the compound absorbs at either the excitation or emission wavelength. To address this weakness, an alternative gel-based endonuclease inhibitory test was conducted by making use of the hydrolysis property of PA_N_ endonuclease (Fig. 2b). Apparently, our platform (Fig. 2a) enabled the initial identification of PA_N_ endonuclease inhibitors and resulted in the discovery of novel antiviral compounds (Fig. 3a). Therefore, a high throughput screening based on this platform may be feasible when the production of PA_N_ is scaled up.

ANA-0, the analog of PA-30, was highly effective in the treatment of influenza H1N1 virus (supplementary Fig. S2). Since the PA_N_ domain is highly conserved among influenza A subtypes, it was inferred that ANA-0 might be able to provide a broad-spectrum protection against infections by other subtypes of influenza A virus. Our results demonstrated that ANA-0 indeed impeded the replication of different subtypes of the virus, among which the H1N1 and H9N2 strains showed >5 folds of sensitivity than that of the H3N2 and H7N9 strains (Fig. 4a). Alignment on the amino acid sequences by these 4 strains revealed one specific substitutions (V100A) within the PA_N_ domain (supplementary Fig. S1). It was inferred that substitution V100A, when occurred in H3N2 and H7N9, might decrease the binding affinity of ANA-0 to PA_N_, thus the antiviral efficacies varied. *In vivo* study showed that ANA-0 protected mice against lethal challenge of influenza A H1N1 virus (Fig. 5a). Further comparison on the different time points of drug administration revealed that result of 3 or 6 h post-challenge showed better *in vivo* antiviral effect than that of 12 h (supplementary Fig. S3). In addition, there detected >2 log reduction of viral load in the lungs of the ANA-0-treated mice when compared to that of the untreated control group (Fig. 5b). Inflammatory infiltrate and alveolar damage were also largely attenuated in the ANA-0 treated mice (Fig. 5c). These results suggest that ANA-0 has the potential to be developed as an effective anti-influenza therapeutic. Treatments through intranasal route deliver the drug into the influenza virus infection site directly. On the other hand, intranasal administration would significantly facilitate influenza virus infections and promote lung pathology^43^. Therefore, intranasal treatment of influenza virus infections requires several considerations, especially the virus challenge dose and the stress of repeated anesthesia to avoid compromising the effectiveness of a potential antiviral drug^44^,^45^. Taking account of the above factors, as well as the solubility limitation of ANA-0 (i.e. 1 mg/ml in PBS), we chose the therapeutic regimen as described previously. During the submission of this manuscript, one study focusing on the structural and computational analyses of influenza endonuclease inhibitors was published^46^, which might provide valuable information for the further optimization of ANA-0.

The ribonucleoprotein complexes (RNPs) of influenza virus are the independent functional units for viral mRNA transcription and vRNA replication^10^. The viral mRNA transcription is initiated by endonuclease cleavage of 5′-capped RNA fragments from host pre-mRNAs, followed by the elongation and polyadenylation of polymerase activity^11^. Subsequently, the vRNA replication proceeds, which requires the newly synthesized RNP components that are the translation products of earlier step primary mRNA transcription^47^. Since ANA-0 targeted the PA endonuclease domain, it was deduced that the compound should disrupt the virus life cycle by interfering with the initial transcription step. To demonstrate this hypothesis of antiviral mechanism, we first showed that ANA-0 could not inhibit virus entry (Fig. 6a). We then demonstrated that intracellular virus-specific mRNA was significantly suppressed at early stage of ANA-0 treatment, which might result in subsequent reduction of vRNA synthesis (Fig. 6b). The mini-replicon assay result further showed that the virus polymerase activity was impaired in the treatment of ANA-0 (Fig. 6c). The impeded vRNA synthesis may be due to that the progeny vRNPs are the pre-requisites of vRNA replication^48^. As the earlier phase of mRNA transcription impaired, the subsequent steps of protein synthesis and vRNA replication would be abrogated. These results have demonstrated that ANA-0 is an effective inhibitor of viral transcription.

The PA_N_ domain harbors the endonuclease active cavity that is coordinated by the metal binding residues (His-41, Glu-80, Asp-108, and Glu-119), the putative catalytic residue Lys-134, and three strictly conserved residues (Arg-84, Tyr-130 and Lys-137)^49^. The molecular docking results predicted that ANA-0 engaged in the endonuclease active sites and interacted with most of these functional residues (Fig. 7a). In addition, ANA-0 and PA-30 bound tighter to the PA_N_ than that of DPBA (Fig. 7c). This was in line with our primary screening result that ANA-0 and PA-30 exhibited lower IC_50_s in the FRET-based endonuclease inhibitory assay than that of DPBA. Therefore, our results suggest that ANA-0 may function as an optimal endonuclease inhibitor by interacting with the PA_N_ metal binding residues and catalytic residues. Our work underscores the utility of suppressing PA_N_ endonuclease activity as a promising anti-influenza strategy, similar design could be used to develop therapeutics that target to other functional domains of the viral polymerase, e.g. cap-binding domain of PB2 subunit^50^. Besides enzymatic activity, the correct assembly of subunits into functional RdRp is an essential step for influenza vRNA replication. Making use of this strategy, a group of PA C-terminal-targeted compounds were identified to disrupt PA-PB1 interaction with broad anti-Flu activity^51^. In the scenario of increasing drug resistance, combination study between PA_N_-targeted and PA_C_-targeted drugs could be carried out in the future.

Since the antiviral mechanism of ANA-0 was different from the commonly prescribed anti-influenza drug Relenza (zanamivir), we further investigated the antiviral efficacies of drug combinations and demonstrated the synergetic antiviral effect between these two compounds (Table 1). Obviously, therapeutics with synergistically active antiviral compounds provide several advantages over the single-agent treatment, such as enhanced antiviral potency, reduced drug dosage, delayed emergence of drug resistance and fewer side effect^52^,^53^. Importantly, in the circumstance that zanamivir is costly and the frequency of viral resistance to zanamivir is increasing globally^7^,^54^, ANA-0 provides a new addition to the arsenal of anti-influenza treatments.

The propensity of influenza virus to develop resistance to commonly used drugs requires continued development of new therapeutics, especially those are not prone to escape mutation. In this study, we demonstrate the possibility of suppressing viral replication by abrogating the PA endonuclease activity. The established screening platform for endonuclease inhibitors provides new opportunities for the drug discovery. Importantly, the selected compound ANA-0 exhibits substantial potential for clinical applications.

## Methods

### Cells, viruses and chemical compounds

Madin-Darby canine kidney (MDCK) cells were maintained in minimum essential medium (MEM) supplemented with 10% heat-inactivated Fetal Bovine Serum (FBS), 50 units/ml penicillin and 50 μg/ml streptomycin. Upon virus infection, the infected cells were maintained in FBS free media supplemented with 1 μg/ml TPCK trypsin. A total of 8 strains/6 subtypes of influenza A virus, A/HK/415742/09(H1N1), A/Hong Kong/1/1968 (H3N2), A/Shenzhen/406 H/2006(H5N1), A/Hong Kong/156/97(H5N1), A/Vietnam/1194/2004(H5N1), A/Netherlands/219/2003(H7N7), A/Anhui/1/2013(H7N9), and A/HK/1073/1999 (H9N2), were cultured in MDCK cells. A mouse-adapted strain, A/HK/415742Md/09 (H1N1), was propagated in chick embryo. The cultured viruses were titrated by plaque assay and stored at −80 °C in aliquot. All the viruses were conserved by the P3 laboratory, University Pathology Building of Queen Mary Hospital, the University of Hong Kong. All experiments with live viruses were conducted using biosafety level 2 or 3 facilities as described previously^55^. All the testing compounds were purchased from ChemBridge Corporation unless specified.

### Expression and purification of the PA_N_

The PA_N_ domain was expressed in *Escherichia coli* (*E. coli*) and purified by His tag affinity chromatography as described previously^56^. A blank pET32a (+) vector (pET-blank) was transformed, expressed, purified and used as a mock-purified enzyme control.

### FRET-based endonuclease inhibitory assay

To detect the endonuclease activity of the PA_N_, FRET-based endonuclease assay was performed in 96-well black microplates using the method described previously with modifications^18^,^29^. Briefly, a dual-labeled single-strand DNA oligonucleotide (FAM-TCTCTAGCAGTGGCGCC-TAMRA) was synthesized as a detection probe, with FAM (fluorophore) and TAMRA (quencher) conjugated to its 5′ and 3′ terminals, respectively. In a volume of 100 μl, PA_N_ of various concentrations (75, 50, and 25 ng/μl) were incubated with 200 nM DNA probe. Fluorescence signals due to the endonuclease cleavage were measured using VICTOR™ X3 multilabel plate reader (Perkin Elmer) at the wavelength of 485 nm excitation and 535 nm emission. pET-blank protein (75 and 50 ng/μl) and ‘substrate only’ were also included as a mock enzyme control and a background control, respectively. The reaction buffer contained 50 mM HEPES (pH 7.8), 150 mM NaCl and 1 mM MnCl_2._ To validate the specificity, FRET-based endonuclease inhibitory assay was performed. A known inhibitor of influenza endonuclease activity, namely 2, 4-dioxo-4-phenylbutanoic acid (DPBA), was used as a positive control^36^. DPBA was dissolved in DMSO and serially diluted to 100, 25, 6.25, 1.56 μM and incubated with the mixture of PA_N_ (75 ng/μl) and probe (200 nM).

### Gel-based endonuclease inhibitory assay

Alternatively, the endonuclease inhibitory activity of individual compound could be detected by a DNA-gel based assay^56^, in which a single-stranded circular DNA M13mp18 (NEB) was used as the substrate. In a 10 μl reaction volume, 10 μM of each compound was incubated with the mixture of 1 μM of PA_N_ enzyme and 0.2 μg substrate at 37 °C. After 3 h, the reaction was quenched by addition of 20 mM EGTA (Sigma) and final products were loaded for DNA agarose gel electrophoresis and visualized after ethidium bromide staining.

### Small-molecule compounds screening

First, a primary screening of the 950 small-molecule compounds was conducted using the same conditions as described in the FRET-based endonuclease inhibitory assay, in which each compound was tested at the concentration of 10 μg/ml. The positive compounds selected by FRET-based endonuclease inhibitory assay were then verified by gel-based endonuclease inhibitory assay. A dose–response analysis of PA_N_ endonuclease inhibition was then done utilizing both assays. Next, a secondary screening using plaque reduction assay was performed to evaluate the *in vitro* anti-H1N1 efficacies of the identified compounds, in which selected compounds were serial-diluted (10, 5, 2.5, 1.25, and 0.625 μM) and tested. The compounds that showed dose-dependent plaque reductions were selected for further study.

### Selectivity index

Selectivity index (SI) of each compound was defined as the ratio of CC_50_ over IC_50_. CC_50_ value was determined with MTT assay according to the manufacturer’s protocol (Invitrogen), while IC_50_ data was obtained by plaque reduction assay. Both assays were done in MDCK cells and values were calculated using Sigma plot 12.0 (SPSS).

### The evaluation of cross-protection

The antiviral effect of compound ANA-0 and PA-30 were tested against multiple subtypes of influenza virus, including H1N1, H3N2, H5N1, H7N7, H7N9, and H9N2. Briefly, MDCK cells were infected with the viruses at multiplicity of infection (MOI) of 0.002. One hour after virus inoculation, the inoculum was removed and replaced by fresh MEM medium containing 1 μg/ml TPCK trypsin (except H5N1) and serial-diluted compound. The cell-free supernatants were collected at 24 h post-infection and applied to virus titration by plaque assays.

### Assessment of combination treatment *in vitro*

The potential synergistic antiviral effect of ANA-0 and zanamivir (MedChem) was evaluated in cell cultures as described previously^39^. In brief, MDCK cells in 96-well plates were infected with the viruses at low MOI (0.002) for 1 h, washed and then cultured with MEM medium containing zanamivir or ANA-0, alone or in combinations. After 24 h incubation at 37 °C, supernatants of various treatments were collected for virus titration. Five combinations of ANA-0 and zanamivir, at the fixed IC_50_ ratios of 10:1, 5:1, 1:1, 1:5 and 1:10, were included. In each combination, six 2-fold serial dilutions of the stock solution were tested to plot the dose inhibition curve, based on which IC_50_ of individual ANA-0 or zanamivir was determined. Subsequently, fractional inhibitory concentration index (FICI) was calculated using the following formula: FICI = [(IC_50_ of ANA-0 in combination)/(IC_50_ of ANA-0 alone)] + [(IC_50_ of zanamivir in combination)/(IC_50_ of zanamivir alone)]. FICI < 0.5 was interpreted as a significant synergistic antiviral effect^57^.

### *In vivo* evaluation of antiviral effect

BALB/c female mice, 6–8 weeks old, were kept in biosafety level-2 housing and given access to standard pellet feed and water *ad libitum*. All experimental protocols followed the standard operating procedures of the approved biosafety level-2 animal facilities and were approved by the Animal Ethics Committee in the University of Hong Kong. All experiments were performed in accordance with the approved guidelines. After anesthesia, a total of 56 mice in four groups (14 mice/group) were inoculated with LD_80_ (80% lethal dose, 500 PFU/mouse) of mouse-adapted influenza H1N1 virus A/HK/415742Md/09. The therapeutic treatment initiated 6 h post-virus-challenge by intranasal route. Briefly, the mice were anesthetized by intraperitoneal injection of ketamine-xylazine (50/5 mg/kg). Two groups of the mice were treated with ANA-0 and PA-30, respectively. The third group of mice was inoculated with zanamivir as a positive control. The last group was administrated with PBS as an untreated control. Two doses per day of 20 μl of 1 mg/ml of ANA-0 or PA-30 or zanamivir or PBS, i.e. 2 mg/kg/day of each agent were intranasally administered for 3 days. Animal survival and body weight were monitored for 21 days after virus inoculation. A body weight loss of 25% was set as the humane endpoint^58^. Four mice in each group were euthanized randomly on day 4 post-challenge and mouse lungs were collected. Half of lung tissues were frozen for virus titration by standard plaque assay, while half lungs were immediately fixed in 10% buffered formalin for histopathologic analyses as described previously^55^.

### The investigation of antiviral mechanism

MDCK cells were inoculated with influenza H1N1 virus at MOI of 2. To investigate which stage of virus life cycle was interfered by the compound, ANA-0 (20 μM) was added during the time of virus absorption (−1 h) or at 1 h post-infection. For the former, infectious inoculum containing the tested compound was replaced with fresh medium after 1 h incubation; for the later, the compound was maintained in the fresh medium after virus entry. Virus yield in the cells or supernatants was determined at 6 h p.i. by RT-qPCR. To determine the virus replication and transcription upon compound treatment, intracellular viral mRNA and vRNA were detected. After the virus absorption, the inoculum was removed and cells were maintained in the presence or absence of ANA-0 (20 μM). At 3 and 6 h post-infection, cell lysates were collected for RT-qPCR analyses. To obtain the complementary DNA (cDNA) that derived from intracellular viral mRNA or vRNA templates, total RNAs were first extracted from the cell lysates using the RNeasy mini kit (QIAGEN), then reverse transcribed with the first strand cDNA synthesis kit (Roche) using anchored-oligo(dT)_18_ primer (for mRNA) or Uni-12 primer^59^ (for vRNA), respectively. The transcribed cDNA levels were determined by RT-qPCR with specific primers of the HA gene. In the experiments, zanamivir (100 μM) was included as a control of virus release inhibitor. The inhibitory effect of compounds on polymerase activity was evaluated using a mini-replicon assay as described^60^. Briefly, 293 T cells were transfected with 50 ng of each pHW2K-PB1, pHW2K-PB2, pHW2K-PA, pHW2K-NP, pIRES-eGFP (Clontech) and 100 ng of firefly luciferase reporter plasmid pPolI-fluc using Lipofectamine 3000 (Invitrogen). Five hours post-transfection, the medium was replaced by DMEM with 0.5% FBS containing various concentrations (50, 25, 12.5, 6.25, 3.125 and 0 μM) of ANA-0. At 24 h post-transfection, cells were lysed, first applied to measure eGFP fluorescence for transfection efficiency normalization and then applied to luminescence assay using the luciferase reporter assay system (Promega). Both fluorescence and luminescence intensities were measured in a Victor X3 Multilabel plate reader (Perkin Elmer).

### Molecular docking

The structure of PA_N_ bound with DPBA was retrieved from RCSB protein data bank (PDB: 4E5G)^17^. The co-crystallized compound was removed from the structure using PDB editior. Docking analysis was performed with molecular opreting enviornment (MOE) softwere (Chemical Computing Group, Quebec, Canada), considering the protein as a rigid body while ligands were fully flexible. One hundred docking solutions were computed for the ligand-protein interaction and all other parameters were set to default. The best docking score and configuration was selected for further analysis and visualized by Schrodinger maestro (Schrödinger, New York, USA).

### Isothermal Titration Calorimetry

Isothermal titration calorimetry (ITC) titrations were performed with an Auto-iTC200 Isothermal Titration Calorimeter (MicroCal) according to the protocol described by DuBois *et al.*^17^. Data were analyzed using MicroCal Origin 7.0 software using a one-site binding model.

### Statistical analysis

The data were evaluated for statistical significance using one-way ANOVA or Log-rank (Mantel-Cox) test as indicated in the figure legends (Prism 6.0, GraphPad, Inc). Values of *p* < 0.05 were considered to represent a statistically significant difference.

## Additional Information

**How to cite this article**: Yuan, S. *et al.* A novel small-molecule inhibitor of influenza A virus acts by suppressing PA endonuclease activity of the viral polymerase. *Sci. Rep.* **6**, 22880; doi: 10.1038/srep22880 (2016).

## Supplementary Material

Supplementary Information

Supplementary Dataset 1

## Figures and Tables

**Table 1:** Antiviral results of combinational treatments.

Combination Ratio (IC_50_)	IC50 Equivalent^a^		FICI^b^
ANA-0 : Zanamivir	ANA-0	Zanamivir	
10:1	0.25	0.03	0.28*
5:01	0.38	0.08	0.46*
1:01	0.12	0.12	0.24*
1:05	0.07	0.33	0.40*
1:10	0.04	0.35	0.39*

^1^Concentration in IC_50_ equivalent was the normalized concentration that was calculated by dividing the IC_50_ of drug in combination with its IC_50_ alone.

^2^FICI was the sum of ANA-0 and zanamivir IC_50_-equivalent concentrations used in each combination;

^3^FICI < 0.5 was interpreted as significant synergistic effect.
