# Supplementary material for: A novel small-molecule inhibitor of influenza A virus acts by suppressing PA endonuclease activity of the viral polymerase
Source: Sci Rep. 2016 Mar 9;6:22880. doi: 10.1038/srep22880 (PMC4783701; doi:10.1038/srep22880)
Supplement: Supplementary Information [file srep22880-s1.doc]

**Supplementary Information**

**A novel small-molecule inhibitor of influenza A virus acts by suppressing PA endonuclease activity of the viral polymerase**

Shuofeng Yuan^1^, Hin Chu^1^, Kailash Singh^2^, Hanjun Zhao^1^, Ke Zhang^1^, Richard Y. T. Kao^1^, [Billy K. C. Chow](http://link.springer.com/search?facet-author="Billy+K.+C.+Chow")^2^,Jie Zhou^1*^, Bo-Jian Zheng^1*^

^1^Department of Microbiology, The University of Hong Kong, Hong Kong SAR, China;

^2^School of Biological Sciences, The University of Hong Kong, Hong Kong SAR, China

***Corresponding authors:**

J. Zhou Tel: +852 22554818; Fax: +852 28551241; Email: [jiezhou@hku.hk](mailto:jiezhou@hku.hk)

B.J. Zheng Tel: +852 22554383; Fax: +852 28551241; Email: bzheng@hkucc.hku.hk


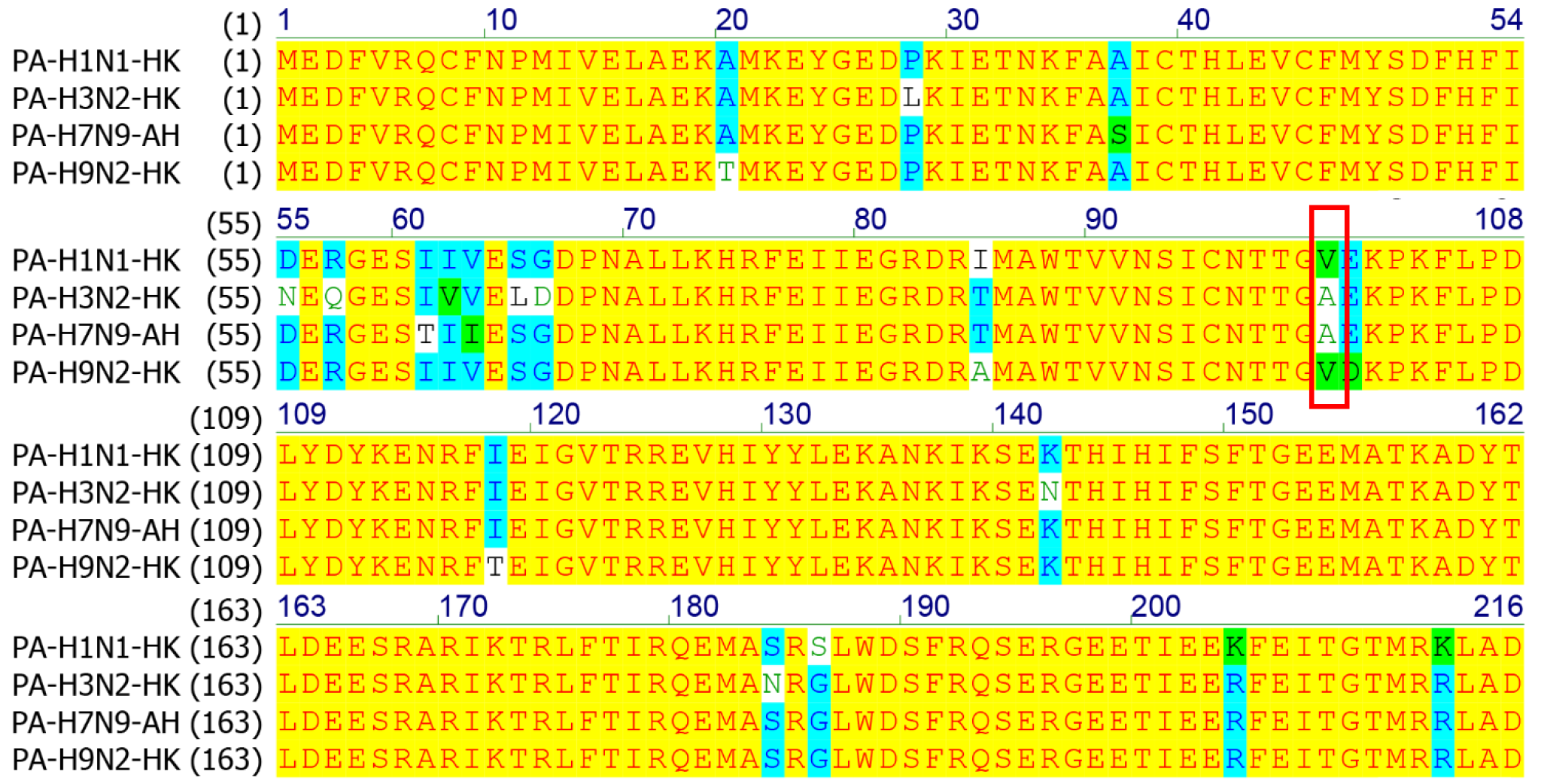


**Supplementary Figure S1.** Sequence alignment of the PA endonuclease domains (residues 1-196) from the influenza H1N1, H3N2, H7N9 and H9N2 virus strains that tested in this study. Residues in a solid yellow background are identical between the four sequences, while substitutions are in cyan or green. The substitution V100A, which is identified in H1N1 and H9N2 strains (i.e.Val-100) but different from that in H3N2 and H7N9 strains (i.e. Ala-100), is highlighted with a red box. The alignment was done by [Vector NTI® Software‎](http://www.google.com.hk/aclk?sa=L&ai=Cu8k1NeGbVfPWJpKA8AW30JH4CN6-lfcG3qKz7ocCm6nxNQgAEAEgjZamGGDZAsgBAaoEIk_QhXjlOdRQUAc5_BFG2W4MzsKyCHiMh5kBT_XcS2cuPPiABZBOgAe23LQjiAcBkAcCqAemvhvYBwE&sig=AOD64_1bZxRd0UBg4G3oR3TWWxpFffNXHg&clui=0&rct=j&q=&ved=0CBgQ0Qw&adurl=http://pixel.everesttech.net/3652/cq%3Fev_sid%3D3%26ev_ln%3Dvector%2520nti%26ev_lx%3Dkwd-113005723%26ev_crx%3D70765703238%26ev_mt%3Db%26ev_n%3Dg%26ev_ltx%3D%26ev_pl%3D%26ev_pos%3D1t1%26ev_dvc%3Dc%26ev_dvm%3D%26ev_phy%3D9061607%26ev_loc%3D%26url%3Dhttp://www.lifetechnologies.com/us/en/home/life-science/cloning/vector-nti-software.html%253Fs_kwcid%253DAL!3652!3!70765703238!b!!g!!vector%252520nti) (Life Technologies).


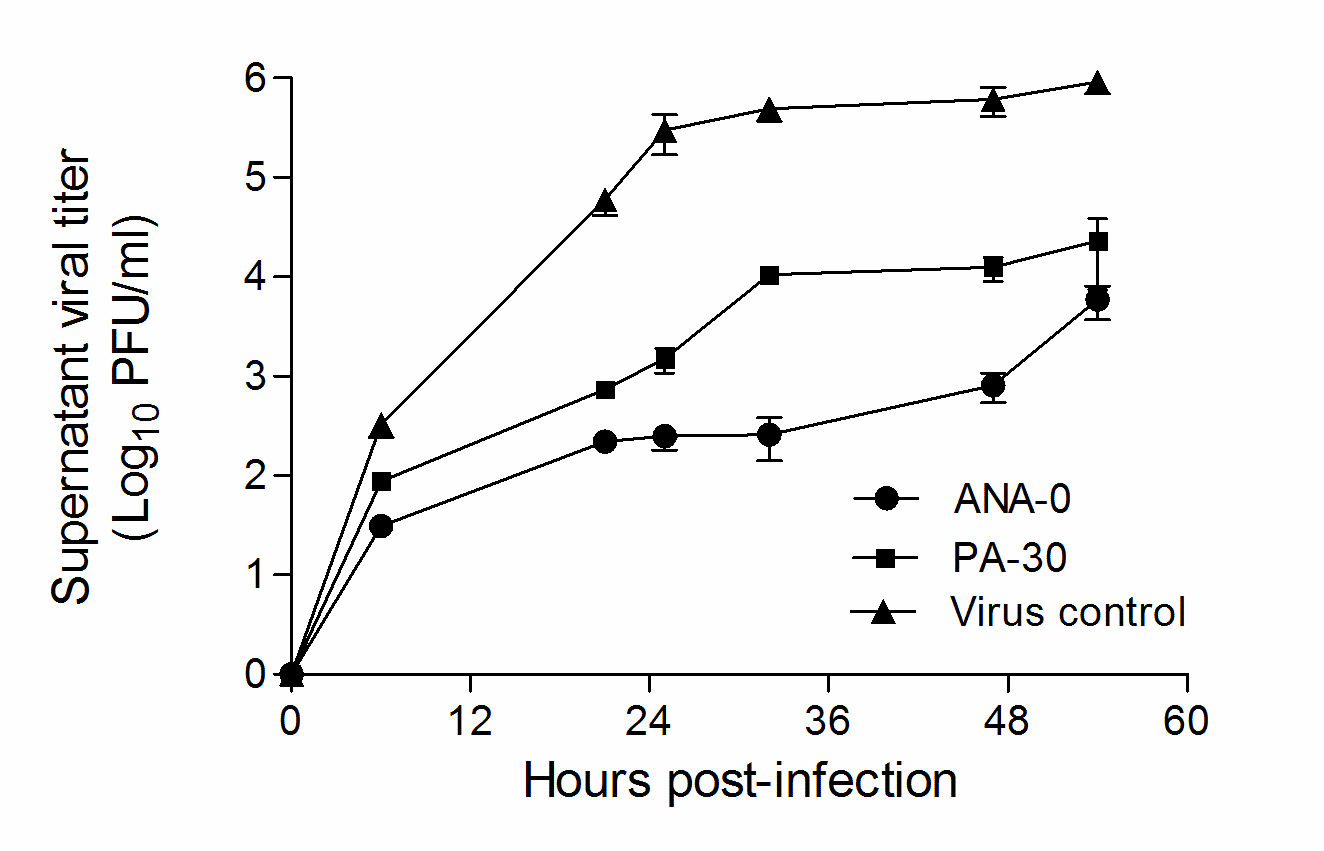


**Supplementary Figure S2.** Antiviral activities of ANA-0 and PA-30 were determined by multi-cycle virus growth assays. MDCK cells were infected with influenza H1N1 virus at MOI of 0.002 and cultured in the presence or absence of ANA-0 or PA-30 (20 µM) after the virus entry. Viral titers in the supernatants were determined by plaque assay at indicated time-points.


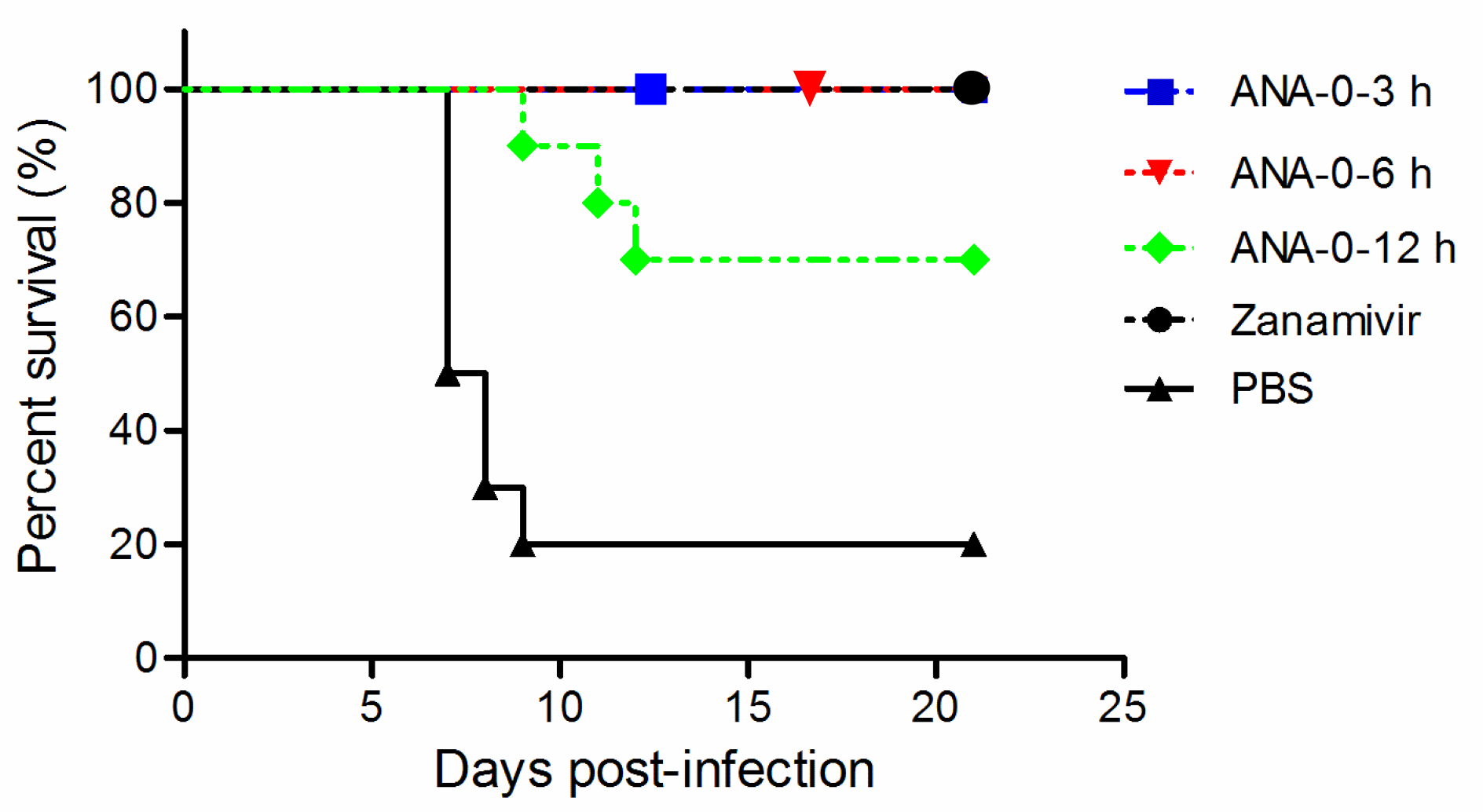


**Supplementary Figure S3.** Efficacy of ANA-0 against H1N1 influenza viruses in mice with different time points of drug administration. Ten mice per group were intranasally infected with 1 LD_80_ of mouse-adapted influenza H1N1 virus A/HK/415742Md/09. Two doses per day of 20 μl of 1 mg/ml of ANA-0, i.e. 2 mg/kg/day were intranasally administered for 3 days following virus infection, beginning 3, 6, or 12 h post-infection. Zanamivir and PBS control groups received equal amounts of treatment with ANA-0, beginning 3 h post-infection. Survival was monitored daily for 21 days.
